# Buprenorphine/naloxone versus methadone opioid rotation in patients with prescription opioid use disorder and chronic pain: study protocol for a randomized controlled trial

**DOI:** 10.1186/s13722-022-00326-1

**Published:** 2022-09-04

**Authors:** Hannah Ellerbroek, Sandra A. S. van den Heuvel, Albert Dahan, Hans Timmerman, Cornelis Kramers, Arnt F. A. Schellekens

**Affiliations:** 1 Department of Psychiatry, Radboud University Medical Center, Nijmegen, The Netherlands; 2 Department of Anesthesiology, Pain and Palliative Medicine, Radboud University Medical Center, Nijmegen, The Netherlands; 3 Department of Anesthesiology, Leiden University Medical Center, Leiden, The Netherlands; 4 Department of Anesthesiology, Pain Center, University Medical Center Groningen, University of Groningen, Groningen, The Netherlands; 5 Department of Clinical Pharmacy, Canisius-Wilhelmina Hospital, Nijmegen, The Netherlands; 6 Department of Pharmacology-Toxicology and Internal Medicine, Radboud University Medical Center, Nijmegen, The Netherlands; 7 Donders Center for Medical Neuroscience, Donders Institute for Brain, Cognition and Behavior, Nijmegen, The Netherlands; 8 Nijmegen Institute for Scientist-Practitioners in Addiction (NISPA), Nijmegen, The Netherlands

**Keywords:** Opioid use disorder, Methadone, Buprenorphine/naloxone, Chronic pain, Current opioid misuse measure

## Abstract

**Background:**

Opioids are effective in pain-management, but long-term opioid users can develop prescription opioid use disorder (OUD). One treatment strategy in patients with OUD is rotating from a short-acting opioid to a long-acting opioid (buprenorphine/naloxone (BuNa) or methadone). Both BuNa and methadone have been shown to be effective strategies in patients with OUD reducing opioid misuse, however data on head-to-head comparison in patients with chronic non-malignant pain and prescription OUD are limited.

**Methods:**

This two-armed open-label, randomized controlled trial aims to compare effectiveness between BuNa and methadone in patients with chronic non-malignant with prescription OUD (n = 100). Participants receive inpatient rotation to either BuNa or methadone with a flexible dosing regimen. The primary outcome is opioid misuse 2 months after rotation. Secondary outcomes include treatment compliance, side effects, analgesia, opioid craving, quality of life, mood symptoms, cognitive and physical functioning over 2- and 6 months follow-up. Linear mixed model analysis will be used to evaluate change in outcome parameters over time between the treatment arms.

**Discussion:**

This is one of the first studies comparing buprenorphine/naloxone and methadone for treating prescription OUD in a broad patient group with chronic non-malignant pain. Results may guide future treatment for patients with chronic pain and prescription OUD.

*Trial registration* https://www.trialregister.nl/, NL9781

## Background

While opioids are potent analgesics on the short-term, evidence to support long-term analgesic efficacy of opioids is weak [1, 2]. Long-term opioid use can result in development of tolerance and therefore reduced effectivity of the medication, requiring increasing doses to sustain the initial level of analgesia [3]. This may lead to dose escalation and development of prescription opioid use disorder (OUD). In patients receiving prescription opioids for chronic non-malignant pain, it is estimated that 21–29% of patients misuses their opioids and 8–12% has an OUD [4]. Other opioid-related side effects include constipation, respiratory depression, cognitive and psychomotor impairments, hallucinations, and opioid-induced hyperalgesia (OIH) [5]. As such, (over)prescribing of opioids is associated with increased morbidities, overdose and mortality [6].

Patients with chronic pain and prescription OUD often experience difficulties in tapering their opioids, due to recurrence of increase of their pain, combined with withdrawal symptoms [7]. An alternative approach to tapering is opioid substitution treatment (OST) from (short-acting) opioids to the long-acting opioids methadone or buprenorphine. Addition of pharmacotherapy to OUD treatment significantly reduces opioid-related harms and increases treatment retention compared to psychosocial treatment without addition of medication [8]. Rotation from short-acting to long-acting opioids commonly serves two goals. First, a switch to long-acting opioids has been shown to reduce the burden of OUD, by reducing withdrawal, craving, and opioid-related harm [9]. Second, switching to long-acting opioids facilitates tapering, because several studies suggest tapering with long-acting opioids might be more successful than with short-acting opioids, especially in case of previously failed tapering attempts [10].

Methadone and buprenorphine are both first-line recommendations for this purpose, mainly studied in OUD patients without chronic pain [11]. Methadone is a full μ-opioid receptor (OR) agonist and NMDA receptor antagonist [12]. Buprenorphine is a high affinity partial μ-OR agonist and kappa-OR antagonist [13]. Buprenorphine is often combined with the μ-OR antagonist naloxone (BuNa), to block μ-OR binding on intravenous use of buprenorphine [14].

It has been suggested that both methadone and buprenorphine have beneficial effects on OIH, although through differential mechanisms [15]. OIH refers to decreased pain thresholds and hypersensitivity to painful stimuli, resulting in an increased pain sensation rather than pain relief [16]. Both the anti-glutamatergic properties of methadone through its effects on the NMDA-receptor [17], and the kappa-opioid receptor antagonizing effects of buprenorphine have been posed to reduce OIH [18, 19]. It is unclear whether methadone and buprenorphine differ in their effects on OIH in patient with chronic pain and OUD.

The distinct pharmacological profiles of methadone and buprenorphine may have clinical relevance. Though both methadone and buprenorphine have been shown to be effective in reducing opioid-related harm like misuse, overdosing, and mortality, as well as increasing quality of life in patients with illicit OUD [8, 9, 20], patients with illicit OUD showed better retention with methadone than buprenorphine [11, 21–23]. This was mainly observed in the early phase [6 weeks] of treatment [24, 25]. In patients with illicit OUD however, buprenorphine seems to be slightly more effective in reducing misuse of heroin and other drugs [26].

Buprenorphine has a more favorable safety profile than methadone, due to its partial agonism, which results in a ceiling of the effects of opioid and lesser risk for overdose, as well due to its high affinity for the μ-OR, making it competes with other agonists at the μ-OR [13]. Methadone also affects repolarisation time of cardiomyocytes, which has been associated with risk of torsade de pointes [27]. Furthermore, buprenorphine can have a beneficial effect on mood and depression, due to its kappa-OR activity [28–30].

OST with BuNa or methadone is also effective in patients with prescription OUD and chronic pain, and effectively reduces opioid (mis)use and craving while maintaining analgesia [31–36]. Yet, comparative data on BuNa and methadone rotation in patients with chronic pain and prescription OUD are limited. Two small studies by Neumann et al. have compared effectiveness of methadone and BuNa in patients with chronic pain and prescription OUD, but remain inconclusive. One study was terminated prematurely due to misuse of opioids [34]. Both studies were complicated by high drop-out rates of around 50%, and had maximum BuNa dosages at 50% of clinically recommended maximum dosage, potentially confounding study results [33, 34]. Yet, rotation to methadone and buprenorphine showed similar beneficial analgesic effects at 6 months in one study [33], and better analgesia in the methadone group in the other study [34]. Both conditions were associated with improved functioning, fewer cravings, less opioid use and depression, without differences in treatment retention [33, 34]. Despite potentially better analgesia of methadone, the authors recommend BuNa for its favorable safety profile [34]. At our own clinic we recently also rotated 43 patients to BuNa [31, 32]. BuNa treatment was associated with improved analgesia, reduced opioid misuse, fewer cravings, reduced symptoms of depression, anxiety and stress, and higher quality of life compared with pre-treatment. In a Cochrane review, Nielsen et al. also compared substitution treatment with buprenorphine and methadone in patients with pharmaceutical OUD (2 studies [33, 37], n = 155) [26]. The second study, by Saxon et al. [37], is a study with a mixed patient group with patients dependent on illicit opioids and patients dependent on pharmaceutical opioids. Nielsen et al. only included an analysis of the patients dependent on pharmaceutical opioids in their review. The other study included study in the review was the one by Neumann [33]. In the meta-analysis of these two studies, 38% of buprenorphine-treated patients reported substance use compared to 17% of the methadone treated patients, 24 weeks after rotation [26].

Though pharmacologically both rotation strategies might have benefits over the other in terms of safety, adverse effects and analgesia, currently available data cannot guide clinicians when treating chronic pain patients with prescription OUD on the preferred rotation strategy. Given the ongoing (prescription) opioid crisis, this research is urgently needed. The primary aim of the current study is to compare effectiveness between BuNa and methadone rotation in reducing opioid misuse in patients with chronic non-malignant pain and prescription OUD. Secondary aims of this study include assessing domains that are often affected by long-term opioid use, including side effects, analgesia, opioid craving, quality of life, mood symptoms, and cognitive and physical functioning. Based on the data from Nielsen et al. [26], we hypothesize that methadone will be superior to buprenorphine in reducing opioid misuse.

## Methods

### Study design

This study is an open-label randomized controlled trial comparing rotation to methadone with BuNa in chronic pain patients with OUD. Staff collecting data will be blinded, whereas study participants and medical staff will be aware of the intervention.

### Study setting

This study will be performed at the medical psychiatric unit of the Radboud University Medical Center, Nijmegen, The Netherlands. Patients are admitted two to three weeks (flexible, depending on individual situation) for this rotation to allow careful titration of the medication. During this period, patients can participate in daily (social and therapeutic) activities organized at the clinic.

### Study objectives

The primary objective of this study is to compare the effect of rotation to methadone and buprenorphine/naloxone on self-reported opioid misuse 2 months after baseline (prior to rotation). Secondary objectives include comparing the effects of both medications on treatment retention, total prescribed (morphine equivalent) opioid dose, opioid craving, pain intensity, pain sensitivity, quality of life, depression, anxiety, stress, cognitive and physical functioning, and presence and severity of side effects.

### Study population

All patients with chronic non-malignant pain who are referred to the department of psychiatry for treatment of OUD will be asked to participate in this study. We will include one hundred (100) patients.

Participants are eligible if they meet all of the following inclusion criteria; (1) age 18 years or over, (2) meet ICD-11 criteria for chronic non-malignant pain (38), (3) use one or multiple prescribed opioid(s) with a total oral morphine equivalent (OME) dose of at least 60 mg per day for at least 3 months, (4) have an OUD according to the DSM-5 (39), (5) wish to be treated for OUD, (6) are willing to comply to study procedures and (7) are able to give informed consent.

Patients will be excluded it they meet one or more of the following exclusion criteria; (1) current pregnancy, lactating, or planning to become pregnant during the study period, (2) have already used buprenorphine or methadone in the last 4 weeks, (3) use other substances of abuse that prevents safe participation in the study (e.g. severe use of alcohol, benzodiazepines or illicit drugs), (4) have acute psychiatric comorbidity (acute psychosis, acute mania, or severe depression with suicidal ideation), (5) have severe respiratory insufficiency or depression, such as chronic obstructive pulmonary disease GOLD 3 or 4, (6) have serious medical disease, such as severe liver dysfunction (Child–Pugh B or C), severe renal dysfunction (eGFR (MDRD) ≤ 29), heart failure, or current brain trauma, (7) have increased risk for torsade de pointes; a Q-T interval of ≥ 450 ms on an electrocardiograph (ECG), (8) have a hypersensitivity or allergy for buprenorphine, naloxone, methadone, or (9) have another medical or psychiatric condition not specified above that prevents safe participation according to the study physician.

### Study treatments

Patients will be rotated from their current opioid medication to either methadone or BuNa. With either medication, patients are hospitalized to titrate the new medication to a dose appropriate for them (flexible dosing). No placebo will be used, as it has already been established that in patients with OUD rotation to methadone or buprenorphine is more effective than placebo [8, 11]. Also see Table 1 for an overview of the schedule of enrolment, interventions, and assessments.

#### Assignment of interventions

Patients will be randomized into two treatment arms of equal size (2 × 50), using a block design with blocks of varying sizes using the randomization module in CastorEDC [40]. Clinical staff will randomize the patients, while the principal investigator is responsible for eligibility screening.

#### Treatment arm 1: Buprenorphine/naloxone rotation

BuNa (brand name: Suboxone^®^) will be administered in sublingual form. For rotation to BuNa we will use the same dosing protocol as in our previous study [31, 32], which is the standard protocol at our clinic. In patients using long-acting opioids (i.e. extended-release oxycodone), BuNa treatment will only be initiated after switching patients to short-acting oxycodone for 7 days. In patients who only use short-acting opioids, BuNa treatment can be initiated directly. Patients take their last dose of short-acting opioids the evening before rotation. The next day, withdrawal is measured every 4 h using the Dutch versions of the subjective and objective withdrawal scales (SOS and OOS) [41]. Once severity of withdrawal exceeds a total of 12 points out of 132 points on the SOS, treatment is initiated. Patients start with a dose of 4/1 mg BuNa. BuNa is titrated with 2/0.5 mg every 4 h if the patient keeps experiencing withdrawal symptoms (≥ 12 points on SOS). The maximum dose of BuNa is 24/6 mg on the first day of rotation. On the second day, patients receive the entire dosage of the first day. If the patient still experiences withdrawal, additional BuNa is titrated in doses of 4/1 mg. In the following days, dosing will be increased if the patient experiences withdrawal, and decreased if the patient experiences intoxication. After the first 72 h of rotation, dosing can also be increased if pain management is insufficient. The maximum daily dose is 36/9 mg. Once a stable dose has been established, the patient will stay on this scheme until the first evaluation after 1 month. At each monthly evaluation, the dose can be increased or tapered down.

#### Treatment arm 2: Methadone rotation

Methadone will be administered using generic oral methadone tablets. Multiple methods for dosing methadone in non-opioid naive patients have been described in literature, but no consensus has been established on the most effective method [42, 43]. For rotation to methadone, we will use the Dutch guideline for detoxification of psychoactive substances [44]. All full agonist opioids can be directly rotated to methadone. The last dose of the original opioid medication will be taken the evening before rotation. On the first morning of rotation, the first dose will be 25% of the total OME, with a maximum of 30 mg. Depending on withdrawal symptoms (≥ 12 points on SOS, see above), methadone dosage will be increased with 5–10 mg every four hours, to a maximum of dosage of 60 on the first day [44, 45]. In the following days, dosing will be increased if the patient experiences withdrawal, and decreased if the patient experiences intoxication. After the first 72 h of rotation, dosing can also be increased if pain management is insufficient. Once a stable dose has been established, the patient will stay on this scheme until the first evaluation after one month. At each monthly evaluation, the dose can be increased or tapered down.

#### Medical management

Patients will be closely monitored during rotation, and symptoms of withdrawal, pain, or side effects will be treated quickly in line with Dutch guidelines [44, 46]. Withdrawal will be measured with the SOS and OOS, as mentioned above. Pain and side effects will be assessed by patient self-report. Additional medication is available to counteract withdrawal symptoms (clonidine, metoclopramide) and pain (paracetamol, nonsteroidal anti-inflammatory drugs). In case of sleeping difficulties during withdrawal, patients can be supported with melatonin, quetiapine or mirtazapine [46]. Until the first study follow-up at two months post-rotation, the patient is asked to not participate in any other therapies except for those offered at the psychiatric clinic.

### Assessments and outcomes

All questionnaires and study instructions will be given in Dutch. Self-report questionnaires will be sent digitally to the patient prior to their study visits at baseline (t0), and after two (t1) and six (t2) months. All other measures will be conducted in-person.

#### Participant characteristics

The following baseline characteristics are collected at the start of the study (t0);

*Demographic variables; *Age, gender, ethnic background, marital status, educational level, and employment status.

*Medical history; *Duration of pain, location of pain, cause of pain, type of pain, presence of neuropathic pain, presence of illnesses, and surgical history. Presence of neuropathic pain will be assessed using the Douleur Neuropathique 4 (DN4). The DN4 is a 10-item clinician-administered questionnaire. It has 7-interview questions and 3 small sensory tests. Every item is answered with yes (1 point) or no (0 points); a score of ≥ 4 out of 10 indicates the presence of neuropathic pain [47]. In the Dutch population, the DN-4 tool had good reliability and poor-to-moderate validity [48, 49]. We will furthermore collect age of onset of opioid use, duration of opioid use, duration of high-dosed opioid use (OME ≥ 90 mg). OUD will be assessed using the Measurements in the Addictions for Triage and Evaluation Interview (MATE), chapter 4 (dependence and misuse). This is a clinician-administered interview to determine substance use (disorders), commonly used in Dutch addiction care [39].

*Current medication use; *Type and dose of opioid(s), use of non-opioid medication.

*Use of other substances; *Alcohol, tobacco, and drugs. These are measured with the MATE, chapter 1 (substance use).

*Presence of psychiatric diagnoses; *These are measured with the Mini International Neuropsychiatric Interview Plus (MINI-plus). The MINI plus is a standardized, validated, diagnostic interview to assess presence of DSM-4 disorders [50–52].

*Intellectual functioning;* Reading capabilities are measured by the Dutch National Adult Reading test Nederlandse Leestest voor Volwassenen (NLV) [53].

*Influence of reward related cues on one’s actions: Pavlovian to instrumental transfer (PIT); *PIT refers to the interference of Pavlovian conditioning on independently learned instrumental behavior. In alcohol research, higher scores on PIT-tasks have been associated with higher alcohol consumption [54]. In our PIT-task, patients are presented with a computer task. During the Pavlovian conditioning phase, participants are exposed to previously neutral stimuli (images and sounds) that are repeatedly followed by rewards. In a separate instrumental conditioning phase participants are trained to engage in a behavior (pick the right pictures out of a number of pictures) to get rewards. Finally, the irrelevant cues from the Pavlovian phase are presented while engaging in the instrumental task. If the cues from the Pavlovian conditioning phase have been linked to motivational value, they affect the behavioral response [55].

### Primary outcome

This measure is collected at t0, t1 and t2.

*Misuse* is measured by the Current Opioid Misuse Measure (COMM). The COMM is a 17-item self-report questionnaire of aberrant opioid use. It is a reliable and valid tool to monitor opioid misuse in chronic pain patients [56]. Each question has a scale from 0 (never) to 4 (very often) resulting in a total score between 0 and 68 points. The t1 measure is the primary outcome.

### Secondary (explorative) outcomes

The measures are collected at t0, t1 and t2 is measured by the percentage of patients that use their allocated treatment at follow up.

*Pain* is assessed by;

- The visual analog scale for pain (VAS-pain). The VAS-pain consist of a 100-mm line with “no pain” at 0 mm and “worst pain imaginable” at 100 mm, on which patients have to indicate their pain intensity. The VAS-pain is highly correlated with the numeric rating scale for pain [57].
- The Brief Pain Inventory (BPI). The BPI is a self-report questionnaire measuring pain intensity and the extent to which pain interferes with 7 domains of functioning (general activity, mood, walking, normal work, relationships with others, sleep, enjoyment of life). The BPI has been validated for patients with chronic non-malignant pain [58].
- The Central Sensitization Inventory (CSI). The CSI is an indirect measure of central sensitization (a state of hyperexcitability of the central nervous system) [59]. Patients are asked to rate how often they experience certain symptoms, and whether they have been diagnosed with an illness relating to central sensitization (e.g. fibromyalgia, irritable bowel syndrome). In a sample of Dutch chronic pain patients, the CSI had good internal consistency, good discriminative power and excellent test–retest reliability [60].
- (Quantitative) sensory testing (QST). Sensory testing uses sensory stimuli such as cold, electricity and pressure to assess (changes in) pain sensitivity. We include testing of thermal pain thresholds using an ice-water test, and a Tip-Therm stored on ice). Thermal QST can be used to detect opioid-induced-hyperalgesia (OIH) after chronic opioid exposure [74]. We furthermore asses pressure pain thresholds (using the Wagner Force Algometer), electric detection and pain thresholds (using the QST-IV Stimulus Manager from Embedded Control BV.) and sensory detection of a PinPrick and a cotton swab.
- When using standardized procedures, (Q)ST has good intra-observer and test–retest reliability [61].

*Wellbeing *is assessed by;

- A visual analog scale for quality of life (VAS-QOL). The VAS-QOL consist of a 100-mm line with the “lowest imaginable QOL” at 0 mm and the “best imaginable QOL” at 0 mm.
- The World Health Organization Quality of Life Questionnaire- abbreviated version (WHOQOL-BREF). The WHOQOL-BREF is a 26-item questionnaire measuring quality of life. It provides QoL scores on four domains; physical, psychological, social relationships, and environmental QoL [62]. The Dutch WHOQOL-BREF has good validity and reliability [63].
- The Depression, Anxiety, Stress scale (DASS). The DASS is a 42-item questionnaire reporting scores on three subscales (depression, anxiety, stress) and a total score [64]. The DASS has high construct validity and reliability [65].
- A visual analog scale for opioid craving (VAS-craving). The VAS-craving consist of a 100-mm line with “no craving” at 0 mm and “worst imaginable craving” at 100 mm.
- The Global Perceived Effect questionnaire (GPE). This is a one-question questionnaire asking patients to what extent their condition has improved since the start of treatment. The GPE has excellent test–retest reliability, but scores are highly affected by current health status [66].

*Functioning* is assessed by

- The Cognitive Failures Questionnaire (CFQ). The CFQ measures self-reported, subjective cognitive functioning. Patients are asked about the frequency in which they make small errors relating to forgetfulness and absentmindedness [67]. The CFQ has been validated for the Dutch context [68].
- The Montreal Cognitive Assessment (MoCA). The MoCA is a one-page test with tasks on short-term memory recall, working memory, visuospatial abilities, executive functioning, attention, and orientation to time and space [69]. The availability of three different MoCA forms allows for assessment over time [70]. Results on the sensitivity of the MoCA are mixed [71, 72] but it is a useful tool to rapidly screen for presence of (mild) cognitive impairment.
- The Rey Auditory Verbal Learning Test (RAVLT), also known as the 15 Word Test (15WT) [73]. In this test, participants are asked to memorize two lists of 15 nouns. Immediate recall, delayed recall, and word recognition are assessed in a number of different tasks with these two lists. The RAVLT measures changes in memory function over time and has a Dutch version [74].
- The Stroop Color-Word Test (SCWT). The SCWT is a task to measure attention and information processing. In this task, a list of words is presented in different colored fonts. Patients are asked to name all the colors being seen, while ignoring the actual content of the word [75]. Outcomes include the number of error and the Stroop interference (difference between reaction time for congruent and non-congruent combinations of words). Test–retest reliability is acceptable [76].
- The Paced Auditory Serial Addition Task (PASAT). The PASAT is a neuropsychological test and asses several aspects of attention. The primary measure is speed of information processing. PASAT scores are highly affected by age, IQ, and math ability, but is highly sensitive to change over time and has good psychometric properties [77].
- The 6-min walk test (6WT). Patients are asked to walk as fast and as far as they can within six minutes. They may use walking aid or take rests. The total distance walked is the outcome measure. It is an inexpensive test with good reproducibility and is correlated with questionnaires on physical functioning [78, 79].

*Side effects* are measured with the Opioid-Related Symptom Distress Scale (ORSDS). This self-report measure asks whether patients have experienced the most common side effects of opioids (nausea, vomiting, constipation, difficulty passing urine, difficulty concentrating, drowsiness, feeling lightheaded, feeling confused, feelings of fatigue, itchiness, dry mouth, and headache). Frequency, severity and bothersomeness of the side effects are also measured [80].

*Utilization of other medication or therapies* is assessed by self-report. We ask participants about use of concomitant medication and utilization of any other type of therapies, such as pain education, or psychological interventions.

*Opioid dose* is measured at every follow up and is converted to oral morphine equivalent dose (OME).

Next to the COMM, *substance misuse* is assessed by urine-toxicology for buprenorphine, methadone, fentanyl, morphine, oxycodone, tramadol, amphetamines, benzodiazepines, methamphetamines, and cannabis as well as self-report for alcohol and nicotine use.

Data collected for regular care, such as withdrawal severity during rotation, may also be used for scientific analysis.

### Sample size and power analysis

Our primary outcome is to compare mean scores on the COMM two months post rotation (t2) between the BuNa and methadone treatment arms. In our recent study, we rotated 43 patients with chronic pain and prescription OUD to BuNa [31, 32]. In these buprenorphine-rotated patients, the mean COMM decreased by 10 points from pre- to post treatment. Based on pilot data and earlier studies [26], we hypothesize that difference in COMM scores between both treatment groups will be 4.5 points with a standard deviation of 7.5 (effect size d of 0.6). With a two-sided alpha of 0.05, power of 80%, and an allocation ratio of 1:1, we need to include 90 patients. At our inpatient medical psychiatric unit, we currently see a low drop-out of patients. Therefore, we will account for a possible drop-out of 10% and include 100 patients. This sample size calculation was done using a t-test for independent means in G*Power 3.1.9.7 [81].

### Data analysis

Descriptive statistics will be used to summarize demographic variables and to analyze the baseline measures of the primary and secondary outcome parameters. The relationship between the OME of the drug at baseline and the OME of buprenorphine or methadone at follow-up be analyzed using Pearson’s r correlation. The analyses of the primary and secondary parameters will be based on superiority hypotheses and will be conducted with intention to treat analyses. Scores on the primary and secondary study outcomes will be compared between both treatment arms (between subject factor), as well as over time (within subject factor), using a linear mixed model analysis, with the scores on the outcome measures as the dependent factor.

**Table 1:**
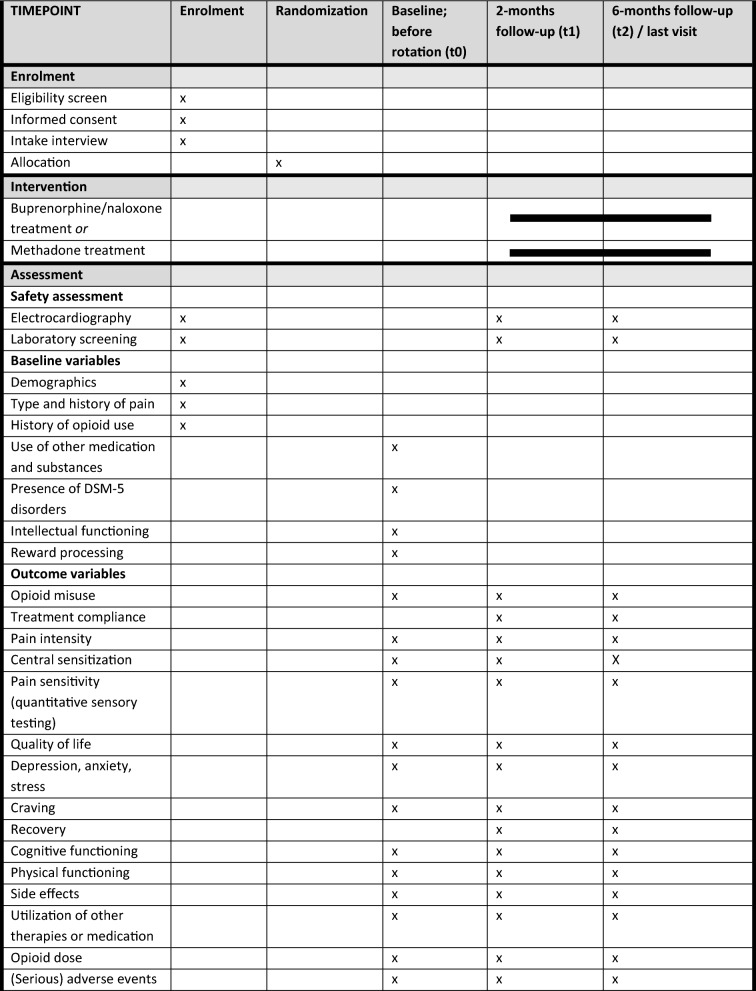
Schedule of enrolment, interventions and assessments

### Data management, monitoring, and safety

For data collection, we utilize electronic case report forms (CRF) in CastorEDC [40], which can be made available upon request. Data will be stored and handled according to the European General Data Protection Regulation. Study monitoring on adequate ethical conduct and compliance with the protocol will follow the guideline for medical studies with negligible risk by the Netherlands Federation of University Medical Centers (NFU) [82]. Participants can discontinue participation in the study (with possibility to continue treatment) anytime. Discontinuation may happen if a patient experiences (severe) side effects or adverse reactions, experiences another (more urgent) medical situation or if they request to stop (with or without disclosure of an explanation). Patients who discontinue the treatment they were assigned to will be considered failures, but will be offered an alternative treatment outside of the study context and in consultation with the study physician. The reason for leaving will be recorded in the CRF and the patient will be asked for consent to use their data until the moment of drop-out. All adverse events will be recorded and all serious adverse events will be reported to the ethical committee without delay.

### Ancillary studies

There will be three ancillary studies to the current one. Participants may or may not choose to participate in these studies without it affecting participation in the main study;

- Evaluating effectiveness of Mindfulness-Based Cognitive therapy (MBCT) as a regular care co-intervention; patients are asked to participate in an 8 week-MBCT training and to fill a few questionnaires to evaluate effectiveness of this intervention.
- Validation study of a 15 SNPs polygenetic test on predicting OUD in the Dutch Setting and explorative study on predictive value of the test for predicting the effectiveness of OST in these patients. Genetic data from patients will be collected by a single cheek-swab at the start of the study. The informed consent form of the main study has a choice whether patients agree in collection and sharing in their genetic data or not.
- Questionnaire validation study. Questionnaires that are used to asses opioid (mis)use will be validated for the Dutch context.

## Discussion

This study will compare effectiveness between methadone and buprenorphine/naloxone on reducing opioid misuse in prescription OUD in patients with chronic non-malignant pain. In a randomized controlled trial, 2 × 50 patients will be randomized to BuNa or methadone treatment. Both medications will be flexibly dosed, to a dose appropriate to the individual patient. The primary outcome is opioid misuse at the two-month follow up. Secondary outcomes include treatment retention, treatment adherence, analgesia, opioid craving, opioid side effects, quality of life, mood symptoms, cognitive and physical functioning at the two- and six months follow-up. Linear mixed model analysis will be used to evaluate change in outcome parameters over time between the treatment arms.

This is one of the first studies directly comparing BuNa and methadone head-to-head in patients with prescription OUD and will provide valuable insights for clinicians. Responses to BuNa and methadone potentially differ in patients with prescription OUD and pain compared to patients with illicit OUD as the provided analgesia is more important for patients with pain. Both buprenorphine and methadone provide analgesia and reduce OIH through differential mechanism. In earlier research, analgesic effectivity of both was close [33, 34, 37].

Individual pharmacokinetics and pharmacodynamics of buprenorphine and methadone will affect individual dosing and effectivity, as bioavailability of both medications is highly intervariable [83, 84]. We will not investigate the pharmacokinetics and pharmacodynamics of both medications, which have already been described in the literature [19, 85], but not yet throughout the course of long-term treatment.

A strength of our study is that we are able to hospitalize our patients for the OST as part of regular medical care. Hospitalization allows for precise titration to a dose appropriate for the individual patient. Buprenorphine has often been dosed insufficiently high in earlier OUD studies [25, 33, 86] increasing risk of withdrawal symptoms and insufficient pain management [23]. Another benefit of hospitalization during titration is that any side effects can be treated rapidly, potentially contributing to treatment adherence.

Another strength of our study is the use of a wide variety of secondary outcome measures to cover a large number of clinical domains that might be important for patients’ well-being. For the pain/analgesia assessment, self-report questionnaires are supported by standardized quantitative sensory testing. Sensory thermal measures are one of the most effective methods to asses hyperalgesia [87].

A limitation of our study is that we compare effectiveness of both medications on a group-level. The current trial will not have enough power to investigate individual predictions of treatment outcome for both medications beyond any explorative analyses, which might be a direction for future studies. Another limitation of our study is the open-label design which may introduce bias. It is not feasible to blind our patients or medical staff, as both medications are administered and titrated individually, and are prescribed to use at home during the follow-up phase. Staff collecting data will however be blinded to prevent bias in data collection.

Despite these limitations, the current study is among the first studies comparing BuNa and methadone treatment in patients with prescription OUD and chronic pain. Results may guide future treatment for patients with chronic pain and prescription OUD.

## Data Availability

Not applicable.

## Abbreviations

15WT: 15 Word test
6WT: 6-Minute walk test
BPI: Brief Pain inventory
BuNA: Buprenorphine/Naloxone
COMM: Current opioid misuse measure
CFQ: Cognitive failures questionnaire
CRF: Case report form
CSI: Central sensitization inventory
DASS: Depression, anxiety and stress scale
DN4: Douleur neuropathique 4
DSM-V: Diagnostic and statistical manual of mental disorders, fifth edition
ECG: Electrocardiograph
eGFR: Estimated glomerular filtration rate
GOLD: Global initiative for chronic obstructive lung disease
GPE: Global perceived effect
ICD-11: International classification of diseases 11th revision
MATE: Measurements in the addictions for triage and evaluation
MBCT: Mindfulness-based cognitive therapy
MDRD: Modification of diet in renal disease study
MINI: Mini international neuropsychiatric interview plus
MoCA: Montreal cognitive assessment
NLV: Dutch national reading test (dutch: nederlandse leestest voor volwassenen)
NMDA: N-methyl-d-aspartate
NWO: Dutch research council
OIH: Opioid-induced hyperalgesia
OME: Oral morphine equivalent
ORSDS: Opioid-related symptom distress scale
OOS: Objective withdrawal scale (Dutch: Objectieve onthoudings schaal)
OR: Opioid receptor
OST: Opioid substitution therapy
OUD: Opioid use disorder
PASAT: Paced auditory serial addition task
PIT: Pavlovian to instrumental transfer
QOL: Quality of life
Q-T interval: On an electrocardiograph: time from start of the Q wave to end of the T wave
QST: Quantitative sensory testing
RAVLT: Rey auditory verbal learning test
SCWT: Stroop color-word test
SNP: Single-nucleotide polymorphism
SOS: Subjective withdrawal scale (Dutch: Subjectieve onthoudings schaal)
t0: Baseline (24 h before rotation)
t1: 2-Month follow-up; t2: 6-month follow-up (last visit)
TAPTOE: Tackling and preventing the opioid epidemic
VAS: Visual analog scale
WHOQOL-BREF: World health organization quality of life—abbreviated version
